# Association of general and central obesity with asthma attacks: A cross-sectional study based on NHANES 2021–2023

**DOI:** 10.12669/pjms.42.9.19822

**Published:** 2026-09

**Authors:** Haoran Zhu, Yuansong Zhang, Xuebo Jiang, Wei Jiang

**Affiliations:** 1 Haoran Zhu, Department of Emergency, The First Affiliated Hospital of Army Medical University, Chongqing 400038, P.R. China; 2 Yuansong Zhang, Department of Emergency, The First Affiliated Hospital of Army Medical University, Chongqing 400038, P.R. China; 3 Xuebo Jiang, Department of Emergency, The First Affiliated Hospital of Army Medical University, Chongqing 400038, P.R. China; 4 Wei Jiang, Department of Emergency, The First Affiliated Hospital of Army Medical University, Chongqing 400038, P.R. China

**Keywords:** Asthma, Asthma attack, Obesity, Waist circumference

## Abstract

**Background & Objectives:**

Obesity has been linked to increased asthma severity, but its direct role in triggering asthma attacks remains unclear. This study examined how general and central obesity are associated with asthma attacks using recent data from the National Health and Nutrition Examination Survey (NHANES).

**Methodology:**

This cross-sectional study analysed NHANES data from August 2021 to August 2023 by the National Centre for Health Statistics for adults aged 18 and older with active asthma. The main outcome was self-reported asthma attacks in the past year. The exposures were body mass index (BMI; categorised as normal, overweight, or obese) and central obesity, defined by waist circumference. Survey-weighted logistic regression accounted for the complex sampling design and adjusted for variables including age, sex, race/ethnicity, education, poverty–income ratio, physical activity, alcohol use, diabetes, and hypertension.

**Results:**

Out of 908 adults with active asthma, 436 reported experiencing at least one attack. Fully adjusted models showed that being overweight was not significantly associated with asthma attacks (adjusted OR 1.14, 95% CI 0.49–2.62), while obesity had a higher but statistically non-significant association (adjusted OR 1.56, 95% CI 0.89–2.72). Central obesity was not independently linked to asthma attacks (adjusted OR 0.95, 95% CI 0.55–1.65). Female sex and hypertension consistently correlated with increased odds of attacks.

**Conclusions:**

Among current U.S. adults with asthma, body measurements alone do not serve as strong independent predictors of asthma attacks after full adjustment, although the results did show a positive trend. The risk of an asthma attack likely involves a complex interplay of social, clinical, and biological factors beyond obesity alone.

## INTRODUCTION

Asthma is a chronic inflammatory condition of the airways that is known to affect people of all ages and is a significant cause of morbidity, increased health service utilization, and reduced quality of life.^1^ There have been advances in pharmacological treatments for the disease in recent decades which have improved symptom control for many patients, however, acute exacerbations of asthma continue to be a significant clinical challenge.^2^ These cause a large number of emergency department visits, hospitalisations, and deaths related to asthma.^3^,^4^ Recent research shows that exacerbations of asthma can be caused by several factors rather than only failure of pharmacotherapy. Evidence suggests that rates of the same are affected by a combination of demographic characteristics, such as social and economic conditions, behaviour, and other comorbidities such as obesity, smoking, acid reflux, and failure to follow treatment plans.^5^,^6^ Guidelines of the Global Initiative for Asthma have shown the importance of identifying and addressing these risk factors to reduce the frequency and severity of exacerbations.^6^ However, the effect of each risk factor can vary significantly across populations, and moreover, the combined influence of multiple factors is not completely understood.^7^

Obesity is a modifiable risk factor affecting both the development and progression of asthma.^8^ Numerous studies have shown that individuals with obesity are more likely to develop asthma, experience more severe symptoms, have poorer disease management, and have more frequent exacerbations than those without obesity.^9^-^11^ Asthma in obese patients is a complex condition which causes changes in lung function, systemic inflammation, metabolic disturbances, and reduces efficacy to inhaled corticosteroids.^12^ While there has been increased research linking obesity and asthma, population-level data on obesity and asthma attacks in current, nationally representative cohorts are limited. The majority of the prior NHANES-based analyses have assessed obesity in relation to asthma prevalence or incidence and not for occurrence of attacks. Also, central obesity has typically been used as a continuous variable, which limits comparability with clinical thresholds used in practice. To the best of our knowledge, this is among the first analyses to examine both general and central obesity using standard clinical cut-points, in relation to asthma attacks using the most recent NHANES 2021–2023 cycle, which reflects the current U.S. adult population following the COVID-19 pandemic. Therefore, the present study aimed to evaluate the association between general and central obesity and asthma attacks among U.S. adults with current asthma, while adjusting for demographic, socioeconomic, lifestyle, and clinical factors.

## METHODOLOGY

This cross-sectional study analysed data from the NHANES August 2021–August 2023 cycle. NHANES is a nationally representative survey of the non-institutionalised U.S. population conducted by the National Centre for Health Statistics. It uses a complex, multistage probability sampling method that includes stratification, clustering, and oversampling of specific subgroups. All participants provide written informed consent.

The present study sample included adults aged ≥18 years with current asthma. Current asthma was defined using two items from the NHANES Medical Conditions Questionnaire: MCQ010 (“Has a doctor or other health professional ever told you that you had asthma?”) and MCQ035 (“Do you still have asthma?”). Participants who answered “Yes” to both items were classified as having current asthma while those answering “No” to either item were excluded. Participants with missing data were excluded through a complete-case analysis to ensure accurate estimates in survey-weighted regression models.

### Ethical approval:

The survey protocols receive approval from the Research Ethics Review Board at the National Centre for Health Statistics. No separate institutional approval was necessary to analyse the data as it is publicly available and de-identified.

### Outcome and exposure:

The primary outcome was the occurrence of an asthma attack. It was defined as self-reported incidence of one or more asthma attacks during the preceding year, as determined from the medical conditions questionnaire. This outcome was converted into a binary variable (yes vs. no). For clarity, the term “asthma attack” is used throughout this manuscript to denote this outcome measure, consistent with the wording of the NHANES questionnaire item; this corresponds to what is more broadly termed an asthma exacerbation in the clinical literature cited in the Introduction and Discussion. We assessed two obesity-related exposures:

General obesity was assessed via body mass index (BMI). BMI categories included: normal (<25 kg/m²; reference group), overweight (25.0–29.9 kg/m²), and obese (≥30 kg/m²).

Central obesity was defined based on sex-specific waist circumference thresholds: 102 cm for men and 88 cm for women. Anthropometric data were obtained as per the standardised physical examinations conducted at mobile examination centres.

### Covariates:

We selected the covariates a priori depending on the clinical relevance and availability of data. These included demographic factors, namely, age (continuous), sex, and race/ethnicity, which were categorised as Non-Hispanic White, Non-Hispanic Black, Hispanic (Mexican American and Other Hispanic combined), and Other race/ethnicity. Socioeconomic indicators included education level and poverty–income ratio. Education was categorised as less than high school, high school graduate, and more than high school, while PIR was analysed as a continuous variable. Lifestyle factors were physical activity and alcohol use which were obtained from questionnaire data and used as binary variables (yes vs no). Clinical comorbidities considered were self-reported physician-diagnosed diabetes and hypertension (yes vs no). Descriptive analyses included laboratory parameters like lipid profiles, glycemic markers, complete blood count, and biochemistry indices. Since complete blood count and biochemistry variables were available only in laboratory subsamples, they were not included in the primary regression models.

### Statistical analysis:

All analyses duly accounted for the intricate design of the NHANES survey, employing the Mobile Examination Centre (MEC) weight (WTMEC2YR), sample strata (SDMVSTRA), and primary sampling units (SDMVPSU) provided in the NHANES demographic file. WTMEC2YR was selected because the exposures of interest, BMI and waist circumference, were derived from anthropometric measurements obtained during the MEC examination. As the August 2021–August 2023 file constitutes a single NHANES release rather than a combination of two separate 2-year cycles, the standard 2-year MEC weight supplied by the National Centre for Health Statistics was applied directly, without additional pooling or rescaling, in accordance with NCHS analytic guidance for this cycle. Continuous variables were summarised using weighted means accompanied by standard deviations, while categorical variables were reported as weighted percentages. Survey-weighted logistic regression analyses were conducted to estimate odds ratios (ORs) and 95% confidence intervals (CIs) for associations between obesity metrics and asthma attacks. Distinct models were developed for BMI categories and central obesity. A sequential modelling approach was adopted, with the fully adjusted model designated as the primary analysis. These models controlled for potential confounders, including age, sex, race/ethnicity, educational attainment, poverty–income ratio, physical activity, alcohol consumption, diabetes, and hypertension. Given the limitations of standard confidence interval estimation in complex survey data, 95% CIs were obtained from model coefficients and robust standard errors derived from the survey-weighted variance–covariance matrix. All statistical procedures were carried out using the R software (survey package), with a two-sided p-value threshold of <0.05 deemed to indicate statistical significance.

## RESULTS

A total of 908 adults with active asthma were enrolled in the study. Study flowchart is presented as Fig.1. Among these, 472 individuals reported no asthma attacks, whereas 436 experienced at least one attack within the past year. The demographic and clinical characteristics of participants, categorized by attack status, are presented in Table-I. Participants who experienced attacks were more likely to be female and to have lower income levels, as indicated by a higher proportion of individuals with low poverty–income ratios. Additionally, differences were observed in race or ethnicity and educational attainment, with a greater number of Hispanic participants and those with less than a high school diploma among the attack group. Comorbid conditions such as hypertension were also more prevalent in this group. Laboratory assessments, including measurements of lipids, blood glucose, hematologic parameters, and other biochemical markers, were summarized and available only for specific sample subsets; therefore, these data were not included in the primary analysis of factors associated with asthma attacks.

**Fig.1:**
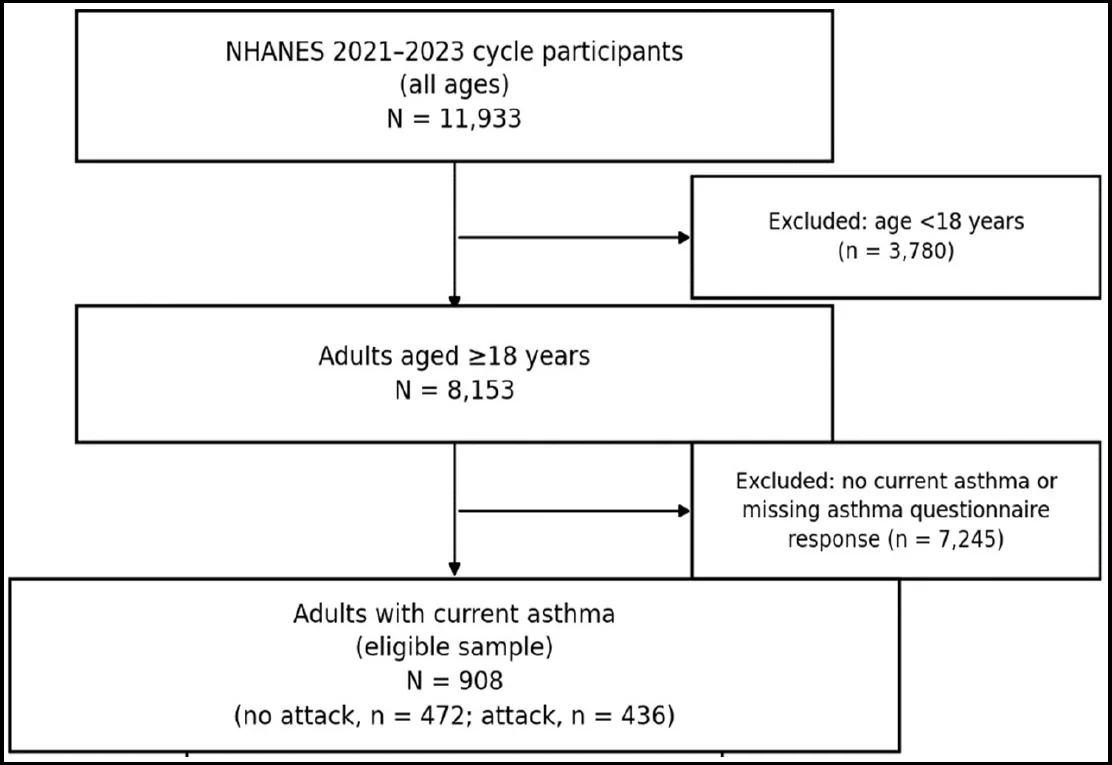
Flowchart of the study.

**Table-I:** Baseline data of participants

Variable	Overall (weighted) (n=908)	No asthma attack (weighted) (n=472)	Asthma attack (weighted) (n=436)
Age (years)	46.7 (18.0)	46.7 (18.9)	46.7 (16.9)
Sex	Male: 39.6%; Female: 60.4%	Male: 48.4%; Female: 51.6%	Male: 30.0%; Female: 70.0%
Ethnicity		Non-Hispanic White: 62.2%	Non-Hispanic White: 58.2%
		Non-Hispanic Black: 14.3%	Non-Hispanic Black: 15.1%
		Hispanic: 10.1%	Hispanic: 14.0%
		Other race/ethnicity: 13.3%	Other race/ethnicity: 12.7%
Education level	>High school: 59.7%	> High school: 61.1%	> High school: 58.3%
	High school graduate: 29.4%	High school graduate: 29.5%	High school graduate: 29.3%
	< High school: 10.9%	< High school: 9.4%	< High school: 12.4%
Poverty–income ratio	2.8 (1.7)	3.0 (1.7)	2.6 (1.7)
BMI (kg/m²)	31.9 (8.9)	30.7 (8.0)	33.2 (9.7)
BMI category	Overweight: 28.8%;	Overweight: 32.1%;	Obese: 55.4%;
	Obese: 49.6%;	Obese: 44.1%;	Overweight: 25.3%;
	Normal: 21.6%	Normal: 23.8%	Normal: 19.2%
Waist circumference (cm)	103.6 (19.0)	102.1 (17.4)	105.4 (20.6)
Central obesity	61.5%	60.4%	62.8%
Smoking status	Former: 58.7%;	Former: 59.7%;	Former: 57.6%;
	Current: 41.3%	Current: 40.2%	Current: 42.4%
Physically active	78.5%	77.7%	79.3%
Alcohol use	86.5%	86.7%	86.3%
Diabetes	15.2%	14.5%	15.9%
Hypertension	39.0%	32.9%	54.3%
HDL cholesterol (mg/dL)	53.8 (15.2)	53.1 (15.2)	54.6 (15.1)
HbA1c (%)	5.8 (1.1)	5.7 (1.0)	5.8 (1.2)
Glucose (mg/dL)	108.5 (32.8)	104.6 (29.9)	112.8 (35.2)
White blood cell count (x 10^9^/L)	7.3 (2.3)	7.1 (2.1)	7.6 (2.4)
Hemoglobin (g/dL)	13.8 (1.5)	13.9 (1.5)	13.7 (1.5)
Platelet count (x 10^3^/µL)	267.9 (74.6)	263.5 (69.0)	272.8 (80.1)
Albumin (g/dL)	4.0 (0.4)	4.1 (0.4)	4.0 (0.3)
Creatinine (mg/dL)	0.9 (0.2)	0.9 (0.2)	0.8 (0.2)
Blood urea nitrogen (mg/dL)	13.8 (4.8)	13.9 (4.9)	13.7 (4.8)

BMI, body mass index; HDL, high-density lipoprotein. Values are survey-weighted using NHANES examination weights (WTMEC2YR) to account for the complex multistage sampling design. Continuous variables are presented as weighted mean (standard deviation), and categorical variables as weighted percentages.Complete blood count and biochemistry parameters were available in laboratory subsamples; therefore, sample sizes for these variables may be smaller than the overall analytic cohort.

### Association between BMI category and asthma attacks:

The relationships between BMI categories and the incidence of asthma attacks are detailed in Table-II. In fully adjusted, survey-weighted logistic regression analyses, overweight status was not significantly linked to asthma attacks relative to normal BMI (adjusted OR 1.14, 95% CI 0.49–2.62). While obesity showed an association with increased odds of asthma attacks, this relationship did not achieve statistical significance after adjusting for demographic, socioeconomic, lifestyle, and cardiometabolic variables (adjusted OR 1.56, 95% CI 0.89–2.72). Among the covariates considered, female sex was independently associated with higher odds of asthma attacks, whereas a higher poverty–income ratio was independently associated with lower odds. Hypertension was also independently linked to increased odds of asthma attacks. Other variables, including race/ethnicity, educational attainment, alcohol consumption, and diabetes, were not significantly associated with asthma attacks in the fully adjusted model.

**Table-II:** Multivariate analysis for BMI as risk factor for asthma attacks

Variable	Adjusted OR	95% CI
Overweight vs Normal	1.14	0.49–2.62
Obese vs Normal	1.56	0.89–2.72
Age (per year)	0.99	0.98–1.01
Female vs Male	2.63	1.75–3.95
Non-Hispanic Black vs NH White	0.82	0.42–1.61
Hispanic vs NH White	1.33	0.68–2.58
Other race vs NH White	1.07	0.51–2.23
High school vs < High school	1.38	0.70–2.72
> High school vs < High school	1.16	0.65–2.08
Poverty–income ratio (per unit)	0.85	0.74–0.98
Alcohol use	0.98	0.47–2.04
Diabetes	0.72	0.42–1.24
Hypertension	1.6	1.06–2.42
***Smoking***		
Former vs Never	0.97	0.69-1.37
Current vs Never	1.18	0.71-1.98

NH, non-Hispanic; OR, Odds ratio; CI, confidence intervals.

### Association between central obesity and asthma attacks:

The link between central obesity and asthma attacks is shown in Table-III. In the fully adjusted, survey-weighted model, central obesity was not independently linked to asthma attacks (adjusted OR 0.95, 95% CI 0.55–1.65). The direction and strength of associations with other covariates in this model were similar to those in the BMI-based model, with female sex and hypertension showing significant associations with increased odds of asthma attacks, while a higher poverty–income ratio was associated with lower odds.

**Table-III:** Multivariate analysis for central obesity as risk factor for asthma attacks.

Variable	Adjusted OR	95% CI
Central obesity (Yes vs No)	0.95	0.55–1.65
Age (per year)	0.99	0.98–1.01
Female vs Male	2.7	1.74–4.20
Non-Hispanic Black vs NH White	0.83	0.44–1.57
Hispanic vs NH White	1.33	0.70–2.53
Other race vs NH White	1.03	0.49–2.16
High school vs < High school	1.31	0.68–2.56
> High school vs < High school	1.14	0.62–2.08
Poverty–income ratio (per unit)	0.85	0.73–0.98
Alcohol use	1.02	0.49–2.11
Diabetes	0.77	0.44–1.36
Hypertension	1.77	1.18–2.65
***Smoking***		
Former vs Never	0.99	0.70-1.41
Current vs Never	1.17	0.72-1.91

NH, non-Hispanic; OR, Odds ratio; CI, confidence intervals.

## DISCUSSION

This cross-sectional study using the NHANES database found no statistically significant independent association between general or central obesity and asthma attacks after adjusting for demographics, socioeconomic factors, lifestyle, and comorbidities. The results for general obesity (BMI ≥30 kg/m²) were directionally consistent with a higher risk of attacks, nevertheless, the association did not reach statistical significance. On the other hand, no such trend was observed for central obesity. This pattern indicates that anthropometric obesity alone may not be a sufficiently powered independent predictor of short-term attacks in this sample, though a modest true association cannot be excluded. The study also found that female sex, hypertension, and lower income were key independent risk factors.

The present study’s findings both align and diverge from prior research on this topic. Several prior studies have examined obesity in relation to asthma prevalence or incidence. A recent meta-analysis by Kong et al.^13^ showed a dose-response relationship between body weight and asthma incidence, finding that overweight individuals had an OR of 1.38 (95% CI 1.17-1.62), while obese individuals exhibited an OR of 1.92 (95% CI 1.43-2.59) for incident asthma compared to normal BMI. Similar findings have been observed in other NHANES-based studies, which have noted higher BMI (>29.9 kg/m²) with a higher prevalence of asthma.^13^ Longitudinal studies have also shown that higher BMI was associated with a greater decline in lung function among overweight and obese individuals.^14^ A NHANES database study on female participants has identified significant nonlinear relationships between BMI and asthma risk. They noted that elevated BMI and higher body roundness index were positively correlated with increased asthma prevalence, which could be due to systemic inflammation, as indicated by higher white blood cell count.^15^ While these studies support a positive association between obesity and asthma prevalence, they primarily reflect the likelihood of developing asthma or having a prevalent diagnosis, rather than the occurrence of attacks among individuals with established disease. Our analysis of asthma attacks did not find statistically significant associations for overweight or obese categories after adjustment for available confounders. Few studies have specifically examined obesity in relation to asthma attacks. A retrospective cohort study in the United States involving 72,086 cases of asthma exacerbation demonstrated that obesity significantly aggravates asthma severity among hospitalised adults by increasing the need for mechanical ventilation. Furthermore, obese asthmatics face a four- to six-fold increased risk of hospitalisation and tend to exhibit poorer disease control.^16^ Similarly, another NHANES-based study has shown that waist circumference was independently associated with a higher risk of asthma attacks, with each 5 cm increase leading to a 6% increased risk, after adjusting for BMI and other covariates.^17^ However, in our fully adjusted models, we noted no significant association between central obesity and asthma attacks. The difference in our results could be due to using waist circumference as a categorical variable to define central obesity rather than a continuous variable.

Obesity can significantly influence the anatomy and physiology of the respiratory system, causing a higher risk of asthma attacks. A primary mechanism involves changes in pulmonary mechanics due to excess adipose tissue. Accumulation of fat around the thoracic wall, abdomen, and diaphragm increases intra-abdominal pressure, restricts diaphragmatic movement, and decreases lung compliance. These mechanical constraints reduce functional residual capacity and expiratory reserve volume, leading to airway closure and ventilation–perfusion mismatch. This, in turn, increases the workload of breathing.^18^,^19^ Such restrictive effects not only diminish lung volumes but also predispose to airway hyperresponsiveness, a characteristic feature of asthma pathophysiology. This is particularly relevant within the small airways, where luminal narrowing can precipitate airflow limitation in response to asthma triggers.^18^ These structural and mechanical alterations are particularly noted in obese individuals and have been thought to contribute to the higher prevalence and severity of asthma.^12^ Beyond such mechanical effects, obesity also leads to a chronic systemic inflammatory state that can change airway biology. Adipose tissue acts as an active endocrine organ, which produces numerous pro-inflammatory cytokines and adipokines. Expansion of adipose tissue, along with cellular stress, hypoxia, and macrophage infiltration, increases circulating levels of inflammatory mediators such as interleukin-6 (IL-6), tumour necrosis factor-alpha (TNF-α), and leptin. Levels of anti-inflammatory adipokines, such as adiponectin, decline in parallel.^11^ These circulating inflammatory signals can reach the pulmonary tissue via the bloodstream leading to airway inflammation or affecting immune responses in the airway mucosa.^8^ Although the specific pathways are unclear, this low-grade inflammatory state may enhance airway reactivity and risk of an asthma attack beyond the mechanical effects of obesity.^8^,^11^ Additionally, obesity-related metabolic disturbances, like insulin resistance and dyslipidaemia, may further contribute to immune dysregulation and affect the pattern of airway inflammation. Some obese patients exhibit a non-eosinophilic, neutrophil-dominant inflammatory profile that may respond differently to standard drug therapies.^18^ Obesity also causes changes in airway smooth muscle responsiveness, extracellular matrix remodelling, and mucus production, which can exacerbate airflow obstruction and increase airway hyperresponsiveness characteristic of asthma.^10^

While BMI and waist circumference are commonly used to assess obesity, they have significant limitations in correctly identifying body fat distribution and the associated metabolic risks. BMI does not distinguish between adipose tissue and lean muscle mass. This can cause misclassification of highly muscular individuals as overweight or obese and the underestimation of adiposity in populations with reduced muscle mass, like the elderly. Moreover, BMI does not provide information on fat distribution, which is a critical factor, as visceral fat surrounding internal organs has different metabolic and inflammatory functions as compared to subcutaneous fat.^20^,^21^ While waist circumference gauges central adiposity, its accuracy is affected by factors such as body posture, respiratory activity, tension of the abdominal musculature, and measurement technique. Furthermore, waist circumference does not differentiate between subcutaneous and visceral fat, nor can it detect ectopic fat deposits in organs such as the liver or muscles.^20^ Both metrics are static indicators that do not account for the dynamic changes in body composition, fat quality, or adipose tissue’s metabolic functions.^20^,^21^ Therefore, reliance solely on BMI and waist circumference may not provide clarity on the more complex relationships between adiposity and health outcomes, especially for asthma.

### Strengths:

It is among the few to evaluate the associations of both general and central obesity in relation to asthma attacks specifically, rather than asthma prevalence or incidence. This distinction is important. Prior obesity-asthma literature has largely examined the risk of developing asthma, whereas clinicians require evidence on which factors predict attacks during ongoing disease.

### Limitations:

Firstly, the cross-sectional nature of the NHANES data cannot allow determination of cause-and-effect relationships, and temporal association between obesity and asthma attacks. Secondly, important variables such as asthma attacks and other covariates were self-reported, which may be affected by recall bias and misclassification. Third, as mentioned earlier, BMI and waist circumference do not accurately reflect body fat distribution, the amount of visceral fat, or the function of adipose tissue, causing incorrect classification of exposure. Variability in waist circumference measurements across different examination settings may have also introduced measurement errors. Fourthly, several clinically important variables could not be included in the adjusted models. Data on medication use like inhaled corticosteroid use, asthma severity classification, healthcare utilisation, and environmental exposures such as allergens and air pollution, were similarly unavailable in this NHANES database. As these factors can independently influence the frequency of asthma attacks and could possibly correlate with body weight, their absence could have led to residual confounding. Therefore, the reported associations should be interpreted with this limitation in mind. Additionally, we used a complete-case approach for the analysis, and participants were excluded for missing data. The included participants may have differed from those excluded, for example in socioeconomic status or disease severity, which could have introduced selection bias and affected the generalisability of the estimates. Nevertheless, an important limitation is that characteristics of excluded versus included subjects were not compared in this study. Lastly, even after adjusting for multiple socioeconomic and clinical factors, several unmeasured or unknown factors were not captured and may have influenced the results.

The results of this study underscore the importance of assessing obesity by means of BMI or waist circumference, when assessing the risk of asthma attacks. While obesity remains a significant and modifiable risk factor, the risk of an asthma attack seems to be affected by a combination of socioeconomic variables, gender, and comorbidities, including hypertension. Therefore, an effective approach to asthma management should include detailed assessment of the patient that includes social vulnerability, comorbid conditions, and access to healthcare alongside traditional clinical and physiological evaluations. Weight management remains a vital component of managing obesity which should be enforced in such patient populations.

### Suggestion:

Given the limitations of the study, further research with prospective designs and larger sample sizes incorporating more data on confounders is needed to clarify the independent contribution of obesity to the risk of asthma attacks. Long-term follow-up studies are also required to determine whether these associations strengthen over longer follow-up.

## CONCLUSIONS

In this NHANES study of adults with asthma, BMI-defined obesity was associated with a positive but statistically nonsignificant link to asthma attacks, although the point estimate indicated a possible positive trend that requires further investigation. However, central obesity was not linked to a higher risk of asthma attacks.

## Recommendations:

Further studies are needed to validate the results.
